# THE EXPERIENCES OF OLDER PEOPLE IN ENGLAND DURING THE COVID-19 PANDEMIC: EVIDENCE FROM ELSA

**DOI:** 10.1093/geroni/igac059.760

**Published:** 2022-12-20

**Authors:** Paola Zaninotto

## Abstract

In this session we will present results from the English Longitudinal Study of Ageing (ELSA) Covid-19 substudy. Data were collected during the pandemic at two separate occasions to capture the experiences of older people and to compare them to pre-covid periods. The session will include results among people with cognitive impairment, people who contracted infections and explore the consequences on their mental health, well-being and health in general

